# Pedogenic controls of soil organic carbon stocks and stability beneath montane Norway spruce forests along a precipitation gradient

**DOI:** 10.1016/j.heliyon.2023.e21284

**Published:** 2023-10-21

**Authors:** Robert M. Bösch, Monika Laux, Walter W. Wenzel

**Affiliations:** University of Natural Resources and Life Sciences, Vienna (BOKU), Department of Forest and Soil Sciences, Institute of Soil Research, Konrad Lorenz Strasse 24, Tulln, A-3430, Austria

**Keywords:** Carbon fractionation, Mean annual precipitation (MAP), Carbonate soil, Ammonium oxalate extractable Al and Fe, Forest soils

## Abstract

Reliable data on SOC stocks in forest soils is required in the context of climate change and soil health assessments but still limited by input data availability (e.g., bulk density) and methods used for stock calculation. Relatively few studies have investigated the stability of SOC in forest soils. We investigated SOC stocks and fractionation in soils beneath Norway spruce forests and grasslands in the montane zone along a gradient of mean annual precipitation (MAP). We sampled soil cores volumetrically to 40 cm depth and measured SOC in the fractions <2 mm (fine earth), >200 μm and 200–20 μm (coarse and fine POM), and <20 μm (MAOM) along with potential pedogenic controls. Total SOC stocks beneath forests in the study region, calculated by the equivalent soil mass (ESM) approach to 40 cm depth, amount to 79.0 ± 29.9 (mean ± standard deviation) Mg ha^−1^ (n = 20) in the mineral soil, and to 92.9 ± 30.6 Mg ha^−1^ including the litter layer, with a share of 55 % associated with POM. MAOM makes up ∼41 % of SOC in the uppermost mineral layer (0–5 cm) and increases to 71 % in the subsoil (20–40 cm). Multiple regression models show that MAOM is largely controlled by ammonium oxalate extractable Al (Al_o_) in the forest subsoils (20–40 cm), and increases with MAP in the topsoil layers (0–20 cm). Soils on carbonate rock stand out with ∼80–100 % larger shares of MAOM in the uppermost soil layers (0–10 cm) which is likely connected to higher soil pH and MAP, supporting microbial transformation and subsequent stabilisation of organic matter, which is reflected in narrower C:N ratios in MAOM and SOC.

Including the litter layers, ESM-based total SOC stocks in forest soils tend to exceed those beneath grassland (80.2 ± 21.9 Mg ha^−1^; n = 31) by 16 %, but only by 6.4 % if calculated by the conventional fixed-depth (FD) approach. In contrast to the forest soils, SOC stocks beneath grasslands are dominated by MAOM (75.6 %).

We conclude that (coniferous) forest soils are a poor reference for establishing sequestration potentials for stable SOC. The observed large proportion of POM in forest topsoils and its increase with declining MAP (indicating water availability) suggests a risk of SOC losses in response to increasing droughts due to climate change.

## 1 Introduction

Globally, soils contain the largest terrestrial C pool [1], and can act as C sink or source, with relatively small changes in SOC having relevant impacts on atmospheric CO_2_ concentrations [2]. In most of the temperate climate zone, forests constitute the natural vegetation cover, and have been considered as ecosystems close to SOC saturation [3,4]. Accordingly, forest soils have been used as benchmarks for estimating the carbon saturation potential of soils, and to derive the C debt (C deficit) of disturbed and managed soils [3].

Wiesmeier et al. [5] identify topography, parent material, soil properties, climate, natural vegetation and soil biota, land use and management as relevant controls of SOC storage, with their relative importance varying in space and time. According to our current understanding of C stabilisation in soils, SOM consists of a continuum of organic fragments that are progressively processed by microbial decomposition towards smaller particles and decreasing molecular size from large biopolymers towards monomers [6]. Stabilisation of SOC is related to the greater reactivity of smaller, more hydrophilic molecules with mineral surfaces and their enhanced incorporation into soil aggregates [7]. Microbial transformation of plant-derived biomolecules is increasingly viewed as a key process for C stabilisation in soil [8]. As a consequence, soil and climate conditions that favour the microbial transformation of larger SOM fragments are important for SOC stabilisation in soils. As microbial activity largely depends on optimal water availability and temperature, mean annual temperature (MAT) and precipitation (MAP) have been considered as appropriate indicators [5].

In a review of global data, Jobbágy and Jackson [9] report a positive effect of MAP on SOC stocks, which is stronger in the uppermost soil layers. Similarly, SOC stocks within each major biome are reported to increase from dry to wet/moist conditions, except in the warm temperate zone [10]. Smaller SOC stocks in dry conditions have been attributed to lower primary production and input of organic materials [5]. A positive relation between water availability and SOM accumulation has also been shown in various studies, e.g., by Hobley et al. [11] at regional scale [5]. In contrast, Fekete et al. [12] recently found a negative relation between MAP and SOC concentrations in soils beneath oak forests in northern and western Hungary, even though surface litter production was smaller in dry conditions. The authors relate this finding to constrained microbial activity and decomposition of organic materials in dry conditions.

In addition to these direct effects of MAP on SOC stocks, precipitation may also control the availability of reactive mineral surfaces. Generally, weathering has been shown to increase with MAP, resulting in accumulation of clay minerals and hydrous oxides of Fe and Al [13,14] that are involved in SOC stabilisation [5]. Carbon storage in soil is also influenced by the mineralogy of the parent material, and has been shown to decrease with increasing silicon concentrations, associated with coarser soil texture and smaller surface reactivity [5].

The physical SOC storage potential of soils has been predicted by the mass fractions of fine soil mineral particles, albeit with different cut offs such as < 20 μm [3,[15], [16], [17]], <50 μm [18], or 53 μm [19]. Apart from the specific surface area provided by fine mineral particles, the SOC stabilisation potential of the mineral phase is determined by the type (e.g., clay minerals versus hydrous oxides of Al and Fe) and quality (e.g., 2:1 versus 1:1 clay minerals) of minerals [6,15].

The proportion of SOC associated with the fine particle fraction is termed mineral-associated organic matter (MAOM) and considered as stable over longer periods, as residence times typically vary between decades and centuries. Conceptually, MAOM is distinguished from labile particulate organic matter (POM) with residence times of a few years to decades. The two SOM pools differ also in chemical composition, with plant- and fungal-derived larger and more complex compounds such as phenols, celluloses and chitins in POM, and smaller, simple low molecular compounds (e.g., amino sugars) of microbial and plant origin in MAOM [20].

Whereas comprehensive data on SOC stocks in forest soils are available for several European regions [[21], [22], [23], [24], [25], [26], [27], [28]], data on SOC pools of different stability are scarce [4]. Moreover, the accuracy of published SOC stock data is partly limited by sampling procedures and the methodology of SOC stock calculations [[29], [30], [31]]. If soils with differential bulk density are compared, the commonly used fixed-depth approach results in overestimation of SOC stocks in soils with high bulk density [32]. This bias can be overcome by the equivalent soil mass (ESM) approach normalizing the SOC stocks to a reference soil mass [30,31]. Employing the ESM approach is of particular importance for the comparison of SOC stocks across different land use systems [33] such as forests and grasslands.

In summary, we conclude that there is a gap of information on unbiased (ESM-based) SOC stocks and pools of differential stability in forest ecosystems. Moreover, our understanding of ecological controls of SOC storage and stabilisation, is still limited and partly inconsistent, calling for additional studies especially at regional scale.

Here we report ESM-based SOC stocks and pools (POM, MAOM) of soils beneath montane spruce forests of Lower Austria that vary primarily in parent material and MAP to explore ecological controls of SOC storage and stability. We benchmark our data to SOC stocks and pools in grassland soils from the same regions, and discuss the implications for using forest soils as C-saturated reference systems.

The following research questions are addressed:

(1) Quantification of SOC stocks and pools of different stability in Central European montane spruce forests and comparison to grassland soils in the same ecological regions;
(2) Does SOC storage and stability in the forest soils of the study region increase (in line with most other studies) or decrease (as found in neighbouring Hungarian regions) with MAP?
(3) Which are the most important soil properties that control SOC storage and stability in the forest soils of the study region?
(4) How do the contributions of MAP and soil controls vary with soil depth?

Based on the literature discussed above, we hypothesize that:

(1) in contrast to the findings along a moisture gradient in neighbouring Hungarian regions [12], the SOC stocks and the share of MAOM in the forest soils increase with MAP;
(2) soil pH, Al_o_, Fe_o_ and the mass of the <20 μm mineral fraction are relevant controls of SOC storage and MAOM [5];
(3) the importance of MAP decreases with soil depth whereas soil controls of SOC storage and stability are more important in deeper layers [5,11];
(4) SOC stocks to 40 cm depth beneath forests do not exceed those of grasslands in the same region, but differ in terms of distribution; MAOM represent a smaller proportion of total SOC stock in forest compared to the grassland soils.

## 2 Materials and methods

### 2.1 Experimental setting and sampling sites of forest soils

We selected four regions with differential climates, bedrocks and soil covers in the Austrian province Lower Austria and identified sampling sites meeting the following criteria:

(1) Elevation above sea level between 450 and 700 m (montane zone)
(2) Norway spruce (*Picea abies* (L.) H. Karst.) forest stands (with a share of silver fir at two sites)

**Fig. 1:**
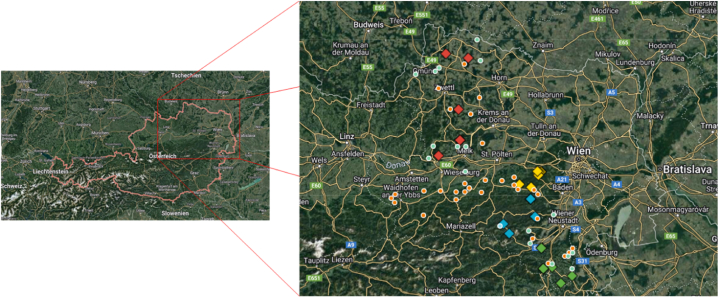
Overview on the study regions and sampling sites in the province Lower Austria. The squares indicate the 20 forest sites, their different filling colours refer to the regions (red, Waldviertel; yellow, Wiener Wald; blue, Kalkalpen; green, Bucklige Welt). The circles with red fillings show the 31 grassland sites used for SOC stock calculations, circles with green fillings the 18 grassland sites for which SOC fractionation of topsoils (0–20 cm) is available. Coordinates are provided in the manuscript data file.

With these limitations we tried to keep litter quality as well as the climate factor MAT in a narrow range while allowing for variation of MAP to explore the effect of water availability on SOC stocks and stability. The sampling locations are shown in Fig. 1. Three regions are dominated by silicate, the fourth region is dominated by carbonate bedrock. The mean elevation of the sampling sites varies among the regions between 511 and 644 m a. s. l., MAT between 7.55 and 8.44 °C, MAP from 705 to 1066 L m^−2^ (Table 1). The climate data for the individual sampling sites were retrieved from the SPARTACUS dataset [34,35] provided by the Institute of Meteorology, University of Natural Resources and Life Sciences Vienna.

**Table 1:** Ecological characteristics of the study regions. Variability measures denote standard deviations.

Geographic region	Code	Geological formation	Type of bedrock	Single tree Ø^a^	MAT^b^	MAP^c^	Elevation	n^d^
				cm	°C	L m-2	m	
Bucklige Welt	SA	Central Alps	Silicates (gneiss, mica schist)	36.6 ± 6.8	8.52 ± 0.55	908 ± 101	606 ± 82	5
Waldviertel	W	Bohemian massif	Silicates (gneiss, granite)	40.6 ± 8.2	7.55 ± 0.43	704 ± 80	644 ± 46	5
Wiener Wald	FZ	Flysch zone	Silicates (sand-, silt, claystone)	31.6 ± 5.9	8.64 ± 0.34	868 ± 33	511 ± 58	6
Kalkalpen	CA	Northern Limestone Alps	Carbonates (limestone, dolomite)	31.6 ± 5.0	8.22 ± 0.12	1066 ± 217	591 ± 56	4

a single tree diameter (at breast height) based on at least 4 trees measured at each individual site.

b MAT, mean annual temperature for the period 1961–2021, data source: Hiebl and Frei (2016).

c MAP, mean annual precipitation for the period 1961–2021, data source: Hiebl and Frei (2018).

d number of sampling sites per region.

According to the World Reference Base for Soil Resources [36], the soils are classified as Dystric and Eutric Cambisols, except soils derived on carbonate rock in the Kalkalpen region (CA), where we sampled Calcaric Cambisols. Detailed classification of all soils is presented in the manuscript data file. The spruce stands are similar in terms of their stage of development, with average single tree diameters varying between 31.6 and 40.6 cm among the regions.

### 2.2 Soil sampling and preparation

In each region, we collected three soils at one of the sampling sites to estimate the within-site spatial variability of soil properties. At each of the remaining sites (Table 1), we collected one soil. First, we sampled the organic layer using a wooden frame of 20 × 20 cm, and recorded the mean layer thickness. Subsequently, we collected the mineral soil to 40 cm depth using the split tube sampler set 04.17 (Ejkelkamp, Giesbeek, The Netherlands) with an inner diameter of 5 cm. The sampler was introduced into the soil using a large mallet, gently removed and opened to reveal the mineral soil core. We disassembled the soil core in pre-defined depth increments (0–5 cm; 5–10 cm; 10–20 cm; 20–40 cm) and transferred them in plastic bags.

In the laboratory, we dried the soils at ambient temperature, passed them quantitatively through a 2-mm screen to separate the fine earth (<2 mm) from coarse fragments and roots, and recorded the mass of each fraction.

### 2.3 Grassland soil samples and data

For comparison, we retrieved data for SOC, bulk density estimates and rock fragment contents for the same depth increments of grassland soils from Wenzel et al. [17]. We selected 31 grassland sites located in the same regions within the montane zone (450–700 m a. s. l.). The sampling sites are used for forage production and, to a lesser extent as pastures, with typical coverage levels close to 100 %. Depending on the moisture regime and intensity of use, the grasslands are dominated by the genera *Arrhenaterum*, *Alopecurus*, *Brachypodium*, *Bromus*, *Carex*, *Dactylis*, *Festuca*, *Holcus*, *Lolium*, *Molinia*, *Nardus*, *Sesleria*, or *Trisetum*. For the description of soil sampling, analysis and the calculation of SOC we refer to Ref. [17]. For SOC fractionation as described below, we chose 18 topsoil (0–20 cm) samples representing the study regions, obtained from the Lower Austrian soil inventory archive based at the Institute of Soil Research, BOKU campus Tulln.

### 2.4 Analytical procedures

pH **measurement**. We measured soil pH in a 0.01 M CaCl_2_ suspension after equilibrating for 2 h at a solution-soil ratio of 2.5 mL^−1^ g (mineral soil) or 12.5 mL^−1^ g (organic layers).

**Carbon and nitrogen measurement** [37]**.** We weighed aliquots (15–200 mg) of milled soil in steel vessels and determined the carbon concentrations sequentially at 400, 550 and 900 °C using a soli TOC cube® carbon analyser (Elementar, Langenselbold, Germany, https://www.elementar.com/en/applications/application-explorer/toc-determination-in-mineral-soils-with-the-soli-toc-cube). The sum of the fractions up to 550 °C represents total soil organic carbon (SOC), the carbon released between 550 and 900 °C the inorganic carbon (TIC). Using the stochiometric ratio, we converted the TIC to the CaCO_3_ equivalent. Total nitrogen was detected in the same sample runs using the sTOCcu-conv-TN modul (Elementar, Langenselbold, Germany).

**SOC fractionation**. We measured carbon fractions of differential stability following the procedure of Wenzel et al. [37]. The method physically separates three fractions according to particle size by employing sequential ultrasonic energy and sieving steps. The >200-μm fraction is considered to represent coarse particulate organic matter (coarse POM), either free or associated with macroaggregates, the 200–20-μm fraction fine POM (free or associated with microaggregates), the fraction <20 μm is interpreted as mineral-associated organic matter (MAOM). The stability is deemed to increase in the order coarse POM < fine POM < MAOM.

For breaking up macroaggregates (>200 μm) we placed 30 g sieved (<2 mm), air-dry soil into 200-mL polyethylene containers and added 150 mL deionised water before ultrasonic treatment (Branson W-250 D, Emerson Technologies GmbH & Co. OHG, Dietzenbach, Germany; oscillation 250 Hz, maximal power 200 W) at an output energy of 60 J mL^−1^. The ultrasonic probe was immersed 2 cm into the suspension. Subsequently, we subjected the suspension to wet sieving using a vibration sieving system (Retsch AS 200, 200-μm steel screen; Retsch AG, Haan, Germany) at an amplitude of 50 % and ∼250 mL deionised water to separate the coarse POM fraction. The particles retained in the sieve were rinsed with deionised water until the washing solution was clear, transferred in porcelain dishes and dried to constant weight for at least 24 h at 105 °C. The suspension (<200 μm) passing the sieve was collected in a 2-L glass container. To reduce the volume for the subsequent ultrasonic treatment, the coarser particles were allowed to settle down before the overlying suspension was pumped into a 2-L Schott bottle, and combined with the rinsing solutions. The suspension was passed through a 20-μm screen to separate fine POM, fine sand and silt fractions with an equivalent diameter of 200 - 20 μm. The material retained in the sieve was combined with the sediment of the suspension obtained in the first sieving step, transferred into a clean 200-mL bottle and filled with deionised water to 150 mL; the resulting suspension was cooled down to 5 °C in an ice-filled container to avoid overheating during the subsequent ultrasonic treatment. The output energy of this treatment was set to 440 J mL^−1^ to destroy all microaggregates while avoiding breakdown of silt-sized organic particles. Subsequently, the suspension was subjected to wet sieving through a 20-μm screen. The suspension passing the screen was combined with the one previously pumped into the 2-L Schott bottle, after reducing the volume of the latter. The material retained in the 20-μm sieve as well as the suspension <20 μm were, after reducing the volume, each transferred to porcelain dishes and dried to constant weight for at least 24 h at 105 °C, and the mass of all fractions (>200 μm, 200 - 20 mm < 20 μm) was recorded. The <200-μm fractions were homogenised with a pestle, the >200-μm fraction was milled using a Retsch MM400 mill employing 30 oscillations per second. Subsequently, we measured organic and inorganic carbon in each fraction using a soli TOC cube® carbon analyser as described above.

The mean recovery of the soil mass after fractionation was on average 100 ± 0.83 %. (SD; n = 116); The recovery of the organic carbon determined as the sum of OC in the fractions compared to direct OC measurements of the fine earth (<2 mm) was 87.7 ± 8.76 % (n = 116).

**Specific surface area (SSA).** Following the procedure of Zehetner and Wenzel [38], we equilibrated three g of air-dried soil of the fine fraction (<20 μm) at a relative vapor pressure of p/p_0_ = 0.5273 [39] in a desiccator using a saturated magnesium nitrate hexahydrate (Mg(NO_3_)_2_*(H_2_O)_6_) solution at room temperature for 24 h; after recording the sample masses before and after drying to constant mass at 105 °C we calculated the amount of absorbed water as the mass difference. We calculated the SSA of the fine fraction (m^2^ g^−1^) according to Eq. (1):

(1) $$SSA=\frac{X\;N\;A}{M}$$

where *X* (g g^−1^) is the amount of water absorbed per mass of soil, *N* is Avogadro's number (6.00205 * 10^23^ mol^−1^), *A* is the area occupied per water molecule (1.08*10^−19^ m^2^), and *M* is the molecular mass of water (18.015 g mol^−1^).

**Ammonium oxalate extractable Al (Al**_**o**_**) and Fe (Fe**_**o**_**).** We determined Al_o_ and Fe_o_ in the mineral soil layers using a modification of the Loeppert and Inskeep procedure [40]. After homogenization with a mortar, we placed 0.5 g soil into 100 ml shaking bottles wrapped with aluminium foil, and added 30 mL of 0.175 M ammonium oxalate [(NH_4_)_2_ C_2_O_4_)] + 0.1 M oxalic acid (H_2_C_2_O_4_) solution adjusted with HCl to pH 3. The samples were shaken for 2h in the dark using a rotary shaker and filtered through a filter paper (Grade 1290, Ahlstrom-Munksjö, Helsinki, Finnland). The filtrate was acidified by the same volume of 4 % HNO_3_ and stored at 4 °C. For measuring Al (averaged wavelengths 308.215–396.153) and Fe (238.204–239.562) using ICP-OES (OPTIMA 8300, PerkinElmer, Rodgau-Jugesheim, Germany), we added 1 mL of Y dissolved in 2 % HNO_3_ as an internal standard to the vials containing 10 mL of the acidified filtrate. The reference soil SO26 (subsoil of EUROSOIL 7; [41]) was used as external control.

### 2.5 Calculations and statistics

**Calculations.** All data is presented for oven-dry (105 °C) material. We calculated soil organic matter (SOM) from the measured SOC concentrations by multiplying with the van Bemmelen factor (1.724) and, for the subsequent use as input variable for SOC stock calculations, corrected the recorded fine earth masses (<2 mm) of each depth increment for SOM to obtain the corresponding mineral fine earth mass, which we related to the area (19.63 cm^2^) sampled with the split tube sampler.

We calculated SOC stocks using the equivalent soil mass (ESM) approach [31], employing their R-script for cubic spline fitting to each individual soil profile. The reference soil masses were calculated for each depth increment as the arithmetic means of all 20 forest sites. Their means ± standard errors are 346 ± 23.3 (0–5 cm), 445 ± 25.6 (5–10 cm), 992 ± 48.6 (10–20 cm), and 1967 ± 154 (20–40 cm) Mg ha^−1^ yr^−1^, The reference soil mass for the whole mineral soil (0–40 cm) is 3749 ± 218, that of the 0–20 cm layer 1783 ± 90.1 Mg ha^−1^ yr^−1^. We used the latter also for calculating ESM-based SOC stocks of the grassland soils to allow for unbiased comparison between the land use categories. For comparison, we also calculated SOC stocks using the conventional fixed-depth method by accounting for the volume occupied by rock fragments following the approach of Wenzel et al. [17].

**Statistical analysis.** We processed all statistics in EXCEL Version 16.0 using own computations and standard functions. Prior to t-tests and analysis of variance (ANOVA) we checked the variables for normal distribution using the Kolmogorov-Smirnov test (p = 0.05). If assumptions were met, we employed paired sample *t*-test to evaluate differences between dependent data, and independent-sample t-tests assuming unequal variances otherwise. For comparisons of more than two groups, we used one-way ANOVA to test for the significance of the main effect prior to employing posthoc t-tests to evaluate the significance between individual groups. The significance level of all tests was set to p = 0.05, using test statistics derived from two-tailed hypothesis tests when t-tests were applied.

We further employed single and multiple regression and correlation analysis to explore linear relationships between variables. For regression analysis, we visually checked the prerequisites (Gauss-Markov assumptions) using variable scatter plots, standardised residual plots and Q-Q plots, and, if necessary, log-transformed variables to meet the assumptions.

We prepared all figures and tables in EXCEL 16.0.

## 3 Results

### 3.1 Soil characteristics

The forest soils of the regions dominated by granite, gneiss and mica schists (SA, W; Table 1) are coarse-textured, with mean values of the fine fraction masses (<20 μm) of ∼300–350 g kg^−1^, whereas those derived on sedimentary sand-, silt- and claystone (FZ), or limestone (CA) have a large share of the fine fraction (∼500–550 g kg^−1^) (Table 2). Apart from their similarity in soil texture, the soils of the latter two regions show contrasting SOM contents, with the largest (CA) and smallest (FZ) values of the entire dataset (Table 2). Compared to all other regions, the CA region stands also out in terms of the smallest C:N ratios, higher pH in the slightly acidic to alkaline range, the largest SSA, and considerably smaller Fe_o_ concentrations (Table 2).

**Table 2:** Soil characteristics (means ± standard deviations) beneath spruce forest stands and grassland in the study regions. Region codes: Bucklige Welt (SA), Waldviertel (W), Wiener Wald (FZ), Kalkalpen (CA).

	Unit	Depth increment	Forest soils					Grassland soils
		cm	SA	W	FZ	CA	All regions	All regions
Number of individual sites			5	5	6	4	20	31
Mineral mass fraction <20 μm	g kg^-1^ fine earth	Organic						
		0–5	339 ± 93.8	306 ± 71.9	512 ± 178	526 ± 151	420 ± 158	
		5–10	354 ± 74.4	327 ± 69.2	544 ± 194	568 ± 224	447 ± 179	
		10–20	344 ± 48.2	358 ± 108	540 ± 179	533 ± 297	444 ± 185	
		20–40	330 ± 47.3	355 ± 97.9	532 ± 172	497 ± 325	430 ± 188	
Bulk density (whole soil including coarse fragments)	g cm^-3^	Organic	0.07 ± 0.01	0.11 ± 0.05	0.11 ± 0.03	0.12 ± 0.04	0.10 ± 0.04	
		0–5	071 ± 0.07	0.68 ± 0.22	0.94 ± 0.21	0.66 ± 0.06	0.77 ± 0.21	1.01 ± 0.16
		5–10	0.99 ± 0.06	1.01 ± 0.28	1.24 ± 0.19	1.13 ± 0.23	1.09 ± 0.22	1.19 ± 0.13
		10–20	1.33 ± 0.20	1.18 ± 0.38	1.29 ± 0.09	1.48 ± 0.39	1.31 ± 0.27	1.33 ± 0.07
		20–40	1.43 ± 0.25	1.33 ± 0.20	1.52 ± 0.14	1.65 ± 0.59	1.44 ± 0.37	1.45 ± 0.04
Coarse fragments (>2 mm)	g kg^-1^ whole soil	Organic						
		0–5	129 ± 88.7	68.1 ± 37.4	40.7 ± 36.3	46.3 ± 67.1	70.7 ± 65.6	72.6 ± 76.2
		5–10	290 ± 134	152 ± 81.9	45.5 ± 51.0	230 ± 278	170 ± 166	77.4 ± 76.2
		10–20	346 ± 176	223 ± 160	59.4 ± 63.9	260 ± 257	212 ± 190	100 ± 114
		20–40	414 ± 223	190 ± 94.5	178 ± 183	420 ± 350	288 ± 234	158 ± 147
SOM	g kg^-1^ fine earth	Organic	718 ± 159	693 ± 236	586 ± 185	543 ± 91.8	637 ± 181	
		0–5	124 ± 78.6	123 ± 95.3	69.2 ± 11.7	150 ± 27.7	113 ± 65.8	95.9 ± 35.1
		5–10	59.5 ± 30.2	67.2 ± 53.1	45.6 ± 15.2	93.3 ± 27.7	64.0 ± 35.6	58.5 ± 25.3
		10–20	34.1 ± 18.3	33.0 ± 20.2	27.6 ± 11.5	41.6 ± 14.8	33.4 ± 15.9	34.3 ± 11.7
		20–40	11.3 ± 4.21	16.5 ± 11.1	14.7 ± 5.67	20.8 ± 12.2	15.5 ± 8.52	14.9 ± 6.73
C:N	–	Organic						
		0–5	25.2 ± 6.22	27.0 ± 4.73	18.2 ± 2.08	14.9 ± 2.15	21.5 ± 6.24	10.5 ± 1.89
		5–10	24.5 ± 6.34	26.8 ± 4.95	20.0 ± 1.96	12.5 ± 1.50	21.4 ± 6.40	10.3 ± 9.69
		10–20	29.9 ± 6.34	31.4 ± 7.88	22.9 ± 3.47	13.1 ± 1.16	24.8 ± 8.54	7.46 ± 1.35
		20–40	24.9 ± 6.24	32.0 ± 10.4	18.3 ± 4.22	15.2 ± 1.85	22.7 ± 8.84	6.91 ± 1.66
Specific surface area	m^2^ g^-1^ fine fraction (<20 μm)	Organic						
		0–5	48.1 ± 15.4	57.5 ± 16.0	53.0 ± 15.5	108 ± 38.9	63.8 ± 30.4	
		5–10	42.1 ± 11.4	42.8 ± 10.9	48.6 ± 15.2	104 ± 29.0	56.5 ± 28.9	
		10–20	43.6 ± 10.3	43.0 ± 10.4	47.1 ± 15.8	94.9 ± 30.2	52.6 ± 24.0	
		20–40	43.8 ± 13.5	40.0 ± 9.82	51.8 ± 11.4	107 ± 32.8	55.3 ± 27.7	
pH (0.01 M CaCl_2_)	–	Organic	4.04 ± 0.57	3.77 ± 0.86	4.47 ± 0.85	5.89 ± 0.53	4.47 ± 1.03	
		0–5	3.35 ± 0.23	3.52 ± 0.14	4.12 ± 0.98	6.15 ± 1.37	4.18 ± 1.30	5.70 ± 0.64
		5–10	3.52 ± 0.16	3.67 ± 0.21	4.22 ± 0.97	6.19 ± 1.48	4.30 ± 1.27	5.62 ± 0.70
		10–20	3.76 ± 0.08	3.89 ± 0.25	4.29 ± 0.93	6.67 ± 1.13	4.53 ± 1.30	5.54 ± 0.81
		20–40	3.95 ± 0.16	4.08 ± 0.38	4.43 ± 0.98	7.61 ± 0.31	4.86 ± 1.53	5.66 ± 0.91
Al_o_	g kg^-1^ fine earth	Organic						
		0–5	2.09 ± 0.55	2.93 ± 1.02	1.54 ± 0.58	1.97 ± 0.25	2.11 ± 0.81	
		5–10	1.89 ± 0.45	2.58 ± 0.97	1.53 ± 0.49	1.93 ± 0.35	1.96 ± 0.70	
		10–20	1.61 ± 0.58	2.17 ± 0.80	1.45 ± 0.37	1.85 ± 0.53	1.74 ± 0.61	
		20–40	1.45 ± 0.45	1.72 ± 0.56	1.36 ± 0.37	1.81 ± 0.92	1.55 ± 0.53	
Fe_o_	g kg^-1^ fine earth	Organic						
		0–5	4.27 ± 0.84	4.77 ± 1.26	4.63 ± 2.34	1.83 ± 0.45	4.02 ± 1.80	
		5–10	4.11 ± 1.21	4.05 ± 1.19	4.81 ± 2.42	1.83 ± 0.61	3.85 ± 1.84	
		10–20	3.66 ± 1.18	4.03 ± 1.53	4.48 ± 2.52	1.66 ± 0.91	3.70 ± 1.91	
		20–40	3.16 ± 1.02	3.97 ± 1.62	3.58 ± 1.46	1.48 ± 1.03	3.24 ± 1.49	

Compared to the forest soils of all regions, the grassland soils have higher bulk densities, smaller SOM contents in the upper 10 cm of the mineral soil, and only ∼50 % (v/v) of the coarse fragment content throughout to 40 cm depth. Moreover, the C:N ratios of grassland soils are considerably smaller by ∼50–70 %, and soil pH by ∼0.8–1.5 units higher than beneath forests (Table 2).

### 3.2 SOC concentrations and fractionation

**Fig. 2:**
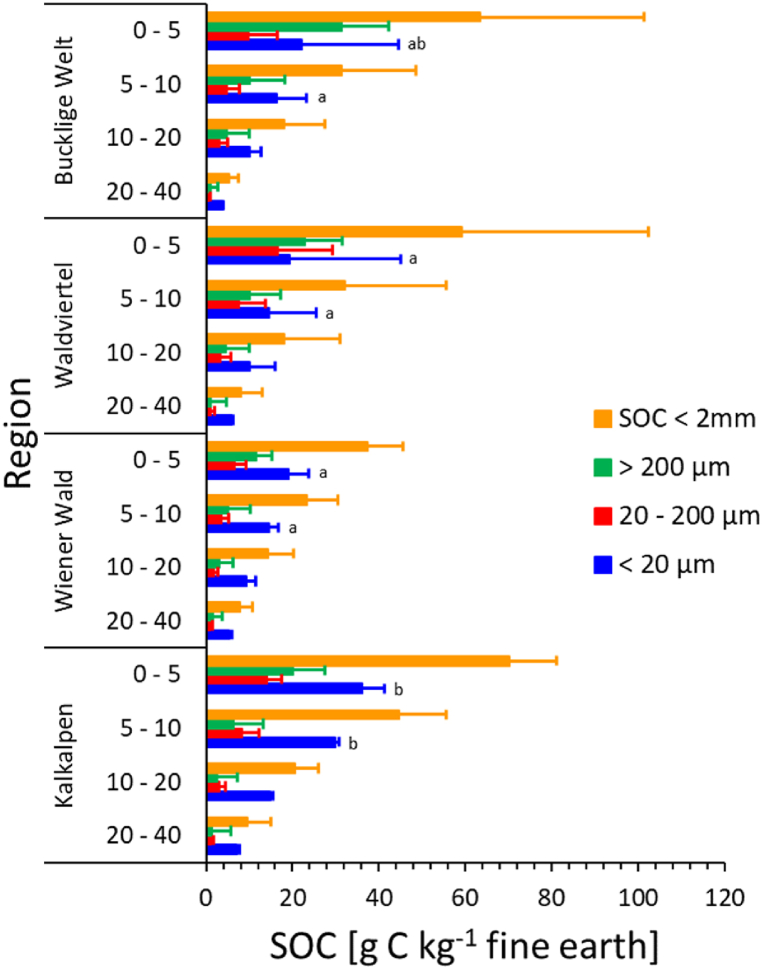
Soil organic carbon (SOC) concentrations in size fractionation of different depth increments of forest soils in the individual regions (number of replicates see Table 1). The graphs show arithmetic means and standard deviations. Significant differences (p < 0.05; t-tests with different variance) within each SOC fraction between regions are indicated by different lowercase letters only for depth increments with significant ANOVA.

The SOC concentrations in different size fractions of the forest soils are shown by depth increment for the four regions in Fig. 2. No significant differences between the regions are observed in the two lowermost soil layers (10–20 and 20–40 cm). In the two upper mineral soil layers (0–5 and 5–10 cm), SOC in the <20 μm fraction (MAOM) is significantly larger by ∼ 80 to > 100 % in the Kalkalpen as compared to all other regions, but no differences are observed in the larger fractions.

**Fig. 3:**
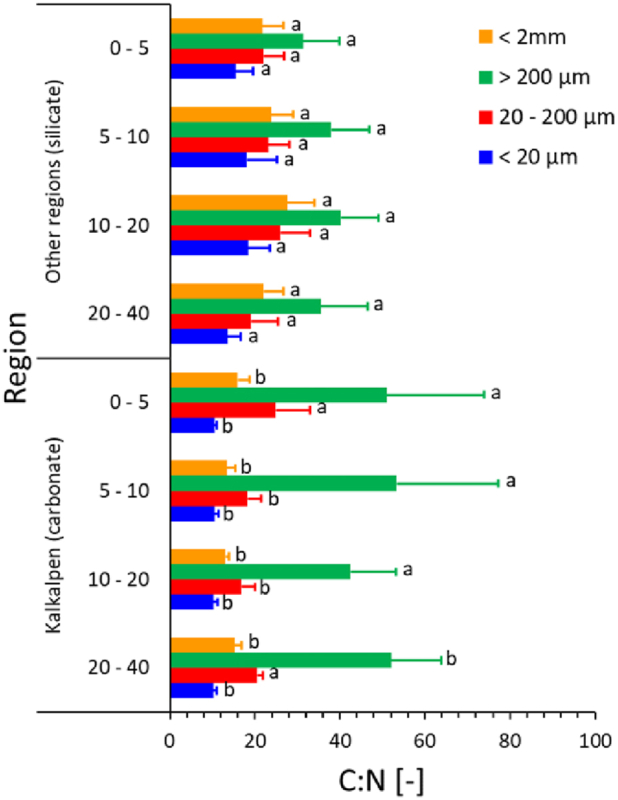
C:N ratios in the SOC fractions in different depth increments of forest soils of the individual regions. The graphs show arithmetic means and standard deviations. Significant differences (p < 0.05; t-tests with different variance) within each SOC fraction between regions are indicated by different lowercase letters.

Fig. 3 compares the C:N ratios in the SOC fractions in the forest soils derived on carbonate bedrock (Kalkalpen) with those developed on silicate parent material in all other regions. The C:N ratios of the fine earth (<2 mm), the fraction 20–200 μm, and MAOM (<20 μm) are significantly smaller in the Kalkalpen as compared to all other regions. In contrast, we find a tendency towards larger C:N ratios in the coarse fraction (>200 μm) in the Kalkalpen region.

**Fig. 4:**
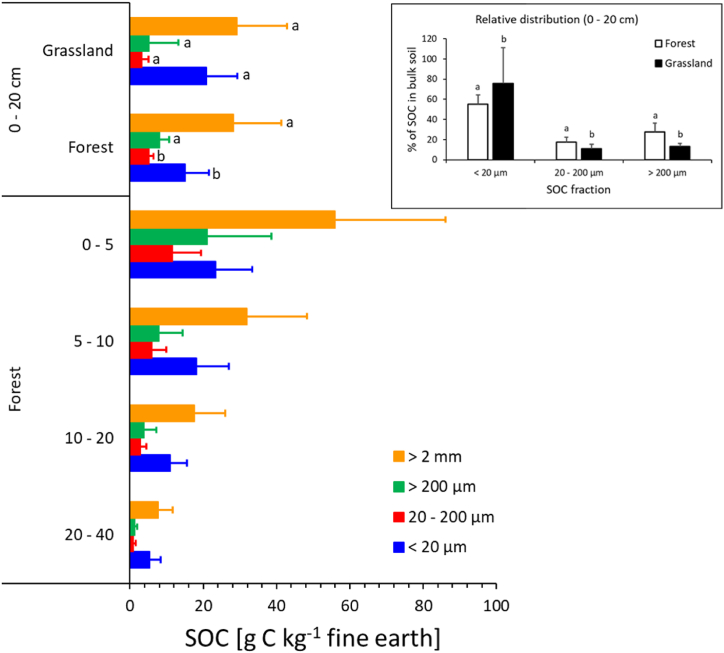
Soil organic carbon (SOC) concentrations in different depth increments of forest soils (N = 20) (bottom), and in grassland (N = 18) versus forest (N = 20) soils (0–20 cm) of the entire study region. The graphs show arithmetic means and standard deviations. Differences between regions are not significant according to one-way ANOVAS performed for each depth increment. The insert shows the relative proportions of individual fractions on bulk (<2 mm) SOC concentrations. Significant differences (p < 0.05; t-tests with different variance) within each SOC fraction between forest and grassland soils are indicated by different lowercase letters.

The arithmetic means of SOC concentrations in the fractions in relation to the fine earth (<2 mm) masses are shown for the entire study region in Fig. 4 (bottom) for each depth increment of the forest soils. The total SOC concentrations in the fine earth are plotted as the sum of the fractions. Along with SOC in the fine earth (<2 mm), the C concentrations of all fractions decrease gradually with soil depth. However, the relative contribution shifts from POM towards MAOM in the deeper layers. Whereas the two POM fractions together dominate in the 0–5 cm increment, the proportion of MAOM exceeds 50 % in the layers below, increasing from ∼41 % (0–5 cm) to 71 % (20–40 cm). POM is made up by ∼54 % by the coarser fraction (>200 μm) in the uppermost layer (0–5 cm) but only by 25–30 % in the deeper layers.

In the upper part of Fig. 4, we compare the SOC fractions in the 0–20 cm mineral soil layer beneath forests and grasslands in the entire study region. To this end, we calculated SOC using the measured concentrations as the mean of the three upper layers, weighed by the soil mass of each layer. We find no significant difference between forest and grassland for the SOC (<2 mm) and the coarse POM fraction (>200 μm) but significantly larger fine POM (200–20 μm) and smaller MAOM (<20 μm) beneath forests (Fig. 4). Expressing the SOC fractions as percentage of the bulk SOC concentrations results for all fractions in significant differences between the two land use categories, with larger shares of both POM fractions but smaller proportion of MAOM in the forest soils (Fig. 4, insert). The average proportion of MAOM in forest soils is only 55.1 ± 9.1 % (median: 57.2 %) which compares to 75.6 ± 13.8 % (median: 80.3 %) in the grassland soils.

### 3.3 SOC stocks

**Fig. 5:**
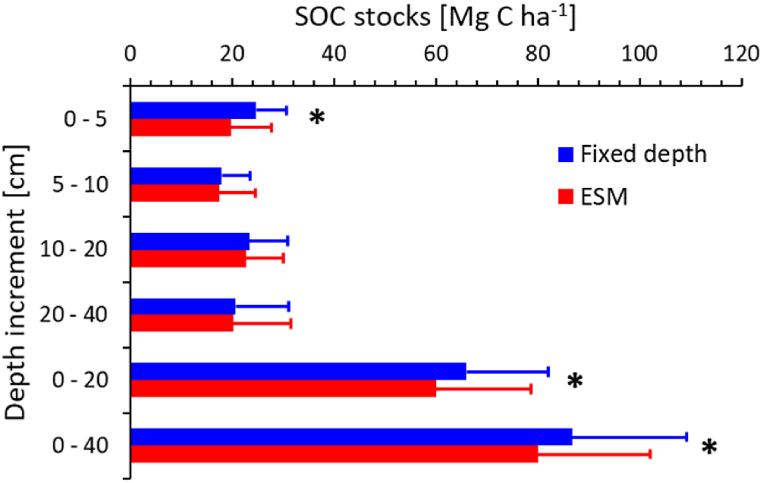
Soil organic carbon (SOC) stocks in different depth increments according to the fixed-depth and equivalent soil mass (ESM, right panel) approach for grassland (N = 31) mineral soils of the entire study region. The graphs show arithmetic means and standard deviations. Reference soil masses are derived from the forest soils of the study region and are presented in the methods section. Significant (p = 0.05) differences between the two methods according to paired t-tests are indicated by asterisk (*).

We calculated SOC stocks following the conventional fixed-depth (FD) and the equivalent soil mass (ESM) approach [31], using the soil masses of the forest soils as reference. The ESM approach allows for more accurate, standardised comparison across soils of different bulk density [30,31]. This is relevant as the bulk densities in the upper layers (0–5 and 5–10 cm) in the grassland soils of our study region are considerably larger than those beneath forests (Table 2). Employing the FD method resulted in ∼8.2 % larger total SOC stocks beneath grassland as compared to the ESM approach (Fig. 5). This difference is significant (p = 0.05) according to a paired *t*-test, and mainly associated with the uppermost mineral soil layer (0–5 cm).

**Fig. 6:**
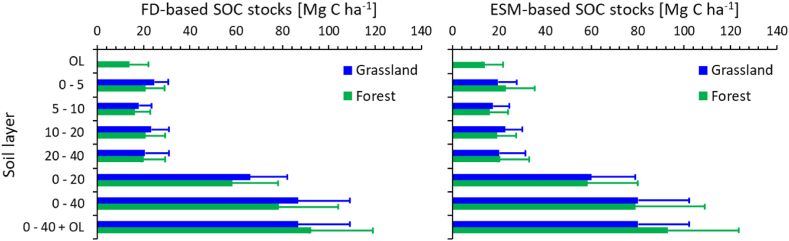
Soil organic carbon (SOC) stocks in different depth increments according to the fixed-depth (FD, left panel)) and equivalent soil mass (ESM, right panel) approach of grassland (N = 31) and forest (N = 20) mineral soils of the entire study region. The graphs show arithmetic means and standard deviations. Reference soil masses for each depth increment are presented in the methods section and are derived as arithmetic means of all forest soils. None of the differences is significant according to a *t*-test with unequal variances (p = 0.05). OL refers to the organic layer.

Both SOC stock calculations approaches reveal similar total SOC stocks and distribution with depth in the forest and grassland soils (Fig. 6). Due to the bulk density bias, the SOC stocks beneath grassland tend to be overestimated by the fixed-depth approach as compared to the ESM-based calculation. The total ESM-based mineral soil stocks to 40 cm depth are almost equal in both land use categories, with 80.2 ± 21.9 (SD) Mg C ha^−1^ in grassland and 79.0 ± 29.9 (SD) Mg C ha^−1^ in forest soils (Fig. 6). As the forest soils contain additional 13.9 ± 8.1 Mg C ha^−1^ in the organic layer, their total SOC stock amounts to 92.9 ± 30.6 Mg C ha^−1^, exceeding that of the grassland soils by ∼16 %, albeit the difference is not significant (p = 0.05) due to the large variation of SOC concentrations among sites within each land use category (Fig. 2).

### 3.4 Correlation and regression models

In an attempt to identify soil and environmental controls of SOC concentrations in the fine earth (<2 mm) and MAOM (<20 μm), we built multiple linear regression models with several relevant soil and climate characteristics as independent variables. Based on available knowledge [5], apart from soil depth, we included the mineral mass fraction <20 μm, pedogenic oxides (Al_o_, Fe_o_) and climate factors (MAT, MAP, or as a surrogate of both, elevation) as independent variables to predict either SOC (<2 mm) or MAOM (<20 μm). The mineral mass fraction <20 μm was only significant (p < 0.05) in the absence of Al_o_ with MAOM as the dependent variable. Neither Fe_o_ nor the molar sum of Fe_o_ plus Al_o_ did add explanatory value in any of the model runs, whereas we identified soil depth and Al_o_ as the primary controls of both MAOM and SOC. The models further improved by including MAP (Table 3).

**Table 3:** Summary of statistical parameters of the multiple linear regression models for explaining SOC concentrations in the fractions <20 μm (MAOM) and <2 mm (fine earth) by soil (depth increment, Al_o_) and climatic (MAP) characteristics. Note that the dependent variables are log-transformed to fulfil the prerequisites of multiple regression analysis. The total number of observations equals 80, corresponding to 20 observations (sites) for each depth increment.

	Regression coefficient	Standard error	P values for regression coefficients	Correlation	ANOVA
log *SOC in fraction <* 20 μm *(MAOM)*					
Intercept	−0.0863	5.0657	8.65 10^-1^		
Depth (cm)	−0.5569	0.0735	7.01 10^-11^		
Alo (mmol kg^-1^ soil)	0.0960	0.0294	1.63 10^-3^		
MAP (mm)	0.0196	0.0046	5.42 10^-5^		
r^2^				0.58997	
Adjusted r^2^				0.57379	
Standard error				6.42307	
Critical F value (ANOVA)					1.035 10^-14^
F value for test statistic					36.54
log *SOC in fraction <* 2 mm *(fine earth)*					
Intercept	1.0220	0.1570	7.32 10^-9^		
Depth (cm)	−0.0226	0.0023	2.60 10^-18^		
Alo (mmol kg^-1^ soil)	0.0047	0.0009	1.79 10^-6^		
MAP (mm)	0.0004	0.0001	3.02 10^-3^		
r^2^				0.75096	
Adjusted r^2^				0.74113	
Standard error				0.19912	
Critical F value (ANOVA)					6.990 10^-23^
F value for test statistic					76.39

**Fig. 7:**
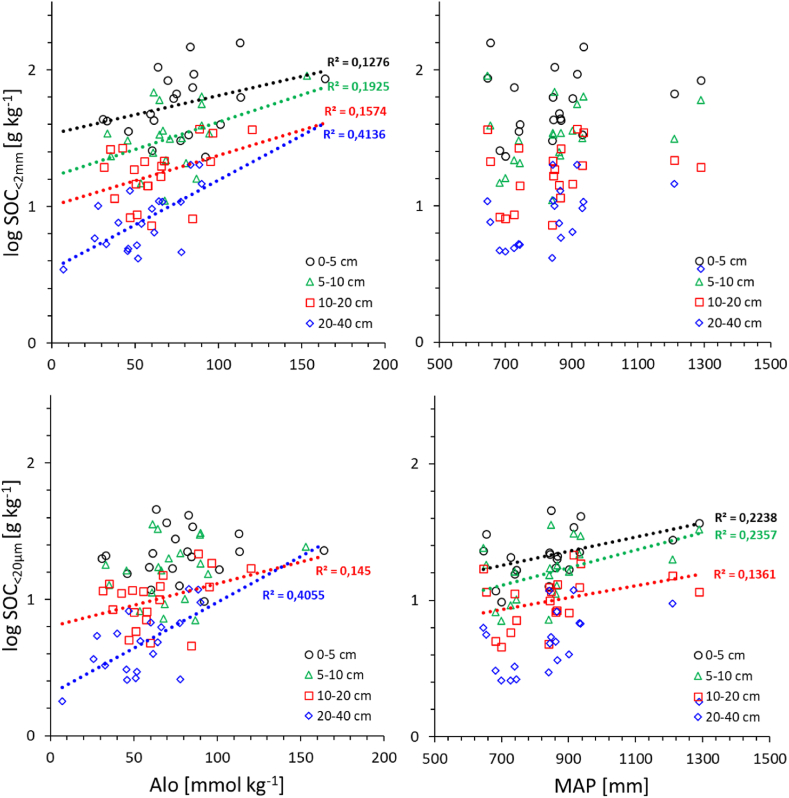
Single linear regression plots of the log SOC in the fine earth (<2 mm) and MAOM (<20 μm) on ammonium oxalate extractable aluminium (Al_o_) and mean annual precipitation (MAP) for different mineral soil depth increments (N = 20 for each relation). Regression lines and goodness of fits (R^2^) are only shown if significant (p < 0.05), and for relations fulfilling the statistical prerequisites of regression analysis.

We also plotted single linear relations between the two significant independent variables (Al_o_ or MAP) and each of the two SOC fractions (Fig. 7). In line with the multiple regression model (Table 3), the plots reveal a stronger influence of Al_o_ as compared to MAP. Moreover, for both SOC fractions the effect of Al_o_ increases whereas that of MAP decreases with soil depth (Fig. 7).

## 4 Discussion

### 4.1 SOC storage and fractionation in forest soils

As expected, the C concentrations of all fractions in the forest soils decrease gradually with depth, whereas the shares of MAOM increase from ∼41 % (0–5 cm) to 71 % (20–40 cm) (Fig. 4). This is well in line with MAOM shares in forest soils of south-eastern Germany increasing from 48.4 % in 0–7 cm to 77.2 % at 25–40 cm depth [42]. Overall, this indicates that the stability of SOM is strongly increasing with soil depth.

We show that SOC concentrations in the <20 μm fraction (MAOM) of the two upper mineral soil layers (0–5 and 5–10 cm) in the Kalkalpen exceeds that of the soils in all other regions (SA, FZ, W; compare Table 1) by ∼ 80 to > 100 % while no differences are observed in the larger fractions (Fig. 2). The soils of the Kalkalpen have been formed on limestone and dolomite rocks whereas the parent material of all other regions is dominated by silicate minerals (Table 1). Moreover, the Kalkalpen region stands out in terms of high MAP, large SSA, small C:N rations and high pH as compared to all other regions (Table 2). This favourable combination of water availability and soil chemical conditions for microbial decomposition is likely to produce a larger share of microbially-derived hydrophilic low-molecular substances, thus enhancing the association of carbon compounds with mineral surfaces and aggregates [[6], [7], [8]]. Moreover, higher soil faunal activity may contribute to SOM accumulation in these soils. This is substantiated by previous reports on positive effects of high soil pH, Ca, Mg and soil moisture on abundance, biomass and functional diversity [43].

The C:N ratios are significantly smaller in the MAOM (<20 μm) fraction at all depths of the soils on carbonate bedrock, indicating the presence of more microbially-derived SOM [19]. In contrast, the larger C:N ratios in the MAOM fraction of the soils formed on silicate parent material exceed the typical microbial range (4.5–15 for fungi, 3–5 for bacteria) [44] and are therefore indicative of a larger share of plant-derived SOM. We also observe a tendency towards larger C:N ratios in the coarse POM fraction (>200 μm) of the soils on carbonate bedrock (Fig. 3) which could be related to selective accumulation of residual plant materials and their *ex-vivo* modification by microbial catabolism to more re-calcitrant compounds in these microbially active soils [8,9].

### 4.2 Environmental controls of SOC storage and fractionation

The results of multiple correlation and regression analysis (Table 3) indicate a strong positive influence of amorphous aluminium oxides (Al_o_) on the SOC concentrations in fine earth (<2 mm) and MAOM (<20 μm). This is consistent with the conceptual model of Rasmussen et al. [45], who, based on statistical evaluation of a large dataset, propose that at low soil pH, organic matter is mainly stabilised by the formation of complexes with Al and Fe, highlighting the importance of their hydrous oxides for SOC storage. Note that the majority of the soils of our study is acidic (Table 2). Whereas in the bulk of previous studies, the fine mineral fraction or clay content have been considered as key variables that determine the physical SOC storage capacity of soils, there is emerging evidence that, depending on pH, other mineralogical characteristics should be considered [45]. For the general study region (Lower Austria), Al_o_ was recently identified as primary soil variable controlling SOC storage and stability beneath hedgerows and adjacent cultivated soils [37].

Apart from Al_o_, only the inclusion of MAP added explanatory value to the multiple regression models (Table 3). Relative to Al_o_, the effect of MAP is small, even though the study was designed along a precipitation gradient, thus emphasising the importance of soil properties as drivers of SOC storage and stabilisation at regional scale [5]. The stronger influence of Al_o_ is also indicated by the single regression models plotted in Fig. 7. They also show that the explanatory value of Alo increased with soil depth whereas that of MAP declines (Fig. 7).

Consistent with previous reports [9,11], our findings reflect the depth-dependent interaction between soil and climate drivers of SOC stabilisation and accumulation. It is reasonable to expect a stronger influence of precipitation in the uppermost soil layers, with related effects on the soil moisture regime. We explain the positive effect of MAP on SOC concentrations in both fractions by improved water availability to soil microorganism, allowing for more effective breakdown of complex biomolecules derived from plant litter and roots, and subsequent stabilisation of smaller, more hydrophilic metabolites of microbial and plant origin on the charged surfaces of the fine mineral fraction [7]. Note that parts of the study region are characterised by relatively dry and warm climate (Table 1), rendering water availability an ecological constraint. A similar MAP gradient starting at, e.g., 1500 mm in combination with lower MAT is therefore likely to shift the climatic control from MAP to MAT.

The stronger influence of MAP in the topsoil layers (0–20 cm) may be also related to the smaller proportion of MAOM (Fig. 4), amounting to 40.3 ± 11.7 % compared to 61.4 ± 7.4 % in the lowest depth increment (20–40 cm). The corresponding larger share of POM near the surface is deemed to be more sensitive to variation in environmental factors and management [19]. A stronger influence of climate drivers on SOC storage in topsoil layers has been also found for global soil datasets by Jobbágy and Jackson [9].

Our finding that SOC concentrations increase with MAP is in line with global [9] and other regional patterns of MAP on SOC storage in soils [11], but in contrast to results of a similar study of SOC in topsoils (0–30 cm) beneath oak forests along a climate gradient in neighbouring regions of Hungary [12]. These authors report almost halved SOC concentrations in the most humid (MAP 725 mm) compared to the driest forest sites (545 mm), along with higher microbial activities (heterotrophic CO_2_ respiration, dehydrogenase activity, fungal biomass) in more humid conditions. We suggest that this apparent contradiction can be explained by the different range of MAP studied by Fekete et al. [12], as their maximum MAP coincides approximately with the lowest MAP of our study. We propose that at severe limitations of water availability, as typical for (forest) steppe climates, SOC concentrations are largely controlled by strong inhibition of microbial activity, resulting in accumulation of plant-derived organic materials with very limited transfer of SOC to stable, slowly cycling pools (MAOM). In the Hungarian study this effect even outperformed the observed decrease in litter production at the dry sites. Our view is supported by modelling results of Fekete et al. [12], indicating shorter turnover times in more humid conditions associated with more efficient transfer from fast to slow cycling SOC pools. Moving to the range of MAP covered in our study (705–1066 mm; Table 1), we expect that, owing to improved water availability, the positive effect of microbial breakdown and transfer to the stable pool (MAOM) on SOC accumulation is more important as droughts become less abundant and severe. Note that the difference in water availability range between the two studies was even more pronounced as the higher MAT (9.6–12.1 °C) at the Hungarian sites promotes higher evaporation rates compared to the Austrian sites (7.6–8.6 °C; Table 1).

The greater importance of the soil mineral phase for SOC stabilisation and accumulation in deeper layers could be related to smaller C:Al_o_ ratios, resulting in stronger C sorption on the less saturated mineral surfaces. Across all soils, the molar C:Al_o_ ratios in the uppermost mineral layer (0–5 cm) are by 1.5–7.3 times larger than at 20–40 cm depth.

### 4.3 Differences between forest and grassland soils

The C:N ratios of both land use categories (Table 2) are close to those reported for topsoils (0–20 cm) beneath coniferous forests (22.5 ± 7.1) and grasslands (11.0 ± 2.1) across Europe [19].

While we find no significant difference between forest and grassland for the SOC (<2 mm) and the coarse POM fraction (>200 μm), forest soils contain larger concentrations of fine POM (200–20 μm) but less MAOM (<20 μm) (Fig. 4). However, the relative shares on SOC (<2 mm) show for all fractions significant differences between the two land use categories, with larger shares of both POM fractions but smaller proportion of MAOM in the forest soils (Fig. 4, insert). The average proportion of MAOM in forest soils is considerably smaller (∼55 %) than in grassland soils (∼75 %). Main factors explaining the lower stability of SOC in forest soils likely include the soil chemical milieu, in particular unfavourably low soil pH (Table 2) for microbial turnover of forest litter material, and its higher persistence. Moreover, in grassland soils C accrual in MAOM may be enhanced due to larger proportions of fine roots, resulting in closer association with mineral surfaces [46,47]. There is also evidence that the dominance of arbuscular mycorrhizae in grassland systems [19] enhances SOC stabilisation by acting as binding agents, stabilising SOC through incorporation in microaggregates [48]. In contrast, coniferous forests such as the spruce forests of our study, are dominated by ectomycorrhiza [9,49].

For Bavarian soils, Wiesmeier et al. [4] found much smaller medians of 60 % for grassland and 38 % for forest soils, whereas an even larger share of 88.5 % MAOM was reported for grassland soils of south-eastern Germany [42]. The proportion of MAOM (<50 μm) was found to be ∼83 % in permanent pastures soils of New Zealand [50] which is close to that for grassland soils in our study. The larger share of POM in forest soils of our region is consistent with a compilation of literature including 13 forest and 22 grassland studies [51], reporting 27.9 % ± 11.3 share of POM for forest soils, and 20.8 % ± 10.9 beneath grasslands.

While the SOC stocks to 40 cm depth in the mineral soils beneath forests and grassland are virtually identical (∼80 Mg C ha^−1^), the total SOC stocks of forest soils are by ∼ 16 % larger owing to the SOC stored in the organic layers (Fig. 6). This finding indicates that in the study region, the historical conversion of forests to grassland had virtually no impact on the total ESM-based SOC stocks in mineral soil down to 40 cm depth. The magnitude of additional storage of SOC in the forest floor is relevant but could be statistically secured only by increasing the sample size. Employing the ESM approach diminished the difference between total SOC stocks of forest and grassland soils from 16 % (FD method) to 6.4 %, supporting the need for reporting ESM-based data, especially if comparisons are made between different land use categories [30,31].

### 4.4 Conclusions

Improving our understanding of SOC stocks and their stability in response to climate and pedogenic controls is important for soil health and climate change mitigation management. In line with previous studies, we show that at regional scale SOC storage and stability is mainly controlled by pedogenic drivers (Al_o_) whereas the effect of the climate factor MAP is significant but less pronounced, and decreases with soil depth.

The quantity of the fine mineral fraction has been extensively used to explain and predict SOC storage and stabilisation [3,5,15,17,19]. However, based on emerging conceptual understanding it has been proposed that other soil physico-chemical variables may be better predictors [5,45]. More recently, pedogenic oxides have been shown to be major determinants of SOC in a wide range of soils at global scale [45] and for specific regions and land use systems [37,52]. Our study expands those findings to temperate forest soils at regional scale. We conclude that apart from relatively high workload and costs [5], the analysis of pedogenic oxides may be worthwhile for improving datasets for the biogeochemical modelling of SOC storage and stability.

Our findings further suggest that increasing droughts due to climate change may pose a risk for SOC loss in the forest topsoils of the study region because of the large share of labile POM, and the observed sensitivity of SOC to MAP.

Finally, we show that the SOC stocks in the mineral soils to 40 cm depth are similar beneath forests and grasslands in our study region whereas the share of stable MAOM is clearly larger in grassland soils. These findings provide evidence that grassland rather than forest soils should be used as reference for determining C saturation potentials of stable C in the soil mineral phase.

## Data availability

All relevant data used to prepare this manuscript is made available in the manuscript data file: Wenzel, Walter; Bösch, Robert; Laux, Monika (2023), “Data file "Pedogenic controls of soil organic carbon stocks and stability beneath montane Norway spruce forests along a precipitation gradient"”, Mendeley Data, V1, https://doi.org/10.17632/tjwghkfm4z.1.

## CRediT authorship contribution statement

**Robert M. Bösch:** Conceptualization, Investigation, Methodology, Project administration. **Monika Laux:** Investigation, Methodology. **Walter W. Wenzel:** Conceptualization, Data curation, Funding acquisition, Methodology, Project administration, Resources, Supervision, Validation, Writing – original draft, Writing – review & editing.

## Declaration of competing interest

The authors declare that they have no known competing financial interests or personal relationships that could have appeared to influence the work reported in this paper.
